# Statistical language learning in neonates revealed by event-related brain potentials

**DOI:** 10.1186/1471-2202-10-21

**Published:** 2009-03-13

**Authors:** Tuomas Teinonen, Vineta Fellman, Risto Näätänen, Paavo Alku, Minna Huotilainen

**Affiliations:** 1 Cognitive Brain Research Unit, Department of Psychology, University of Helsinki, Helsinki, Finland; 2 Finnish Centre of Excellence in Interdisciplinary Music Research, University of Jyväskylä, Jyväskylä, Finland; 3 Department of Pediatrics, University of Helsinki, Helsinki, Finland; 4 Department of Pediatrics, Lund University, Lund, Sweden; 5 Department of Psychology, University of Tartu, Tartu, Estonia; 6 Centre of Functionally Integrative Neuroscience, Aarhus University, Aarhus, Denmark; 7 Department of Signal Processing and Acoustics, Helsinki University of Technology, Espoo, Finland

## Abstract

**Background:**

Statistical learning is a candidate for one of the basic prerequisites underlying the expeditious acquisition of spoken language. Infants from 8 months of age exhibit this form of learning to segment fluent speech into distinct words. To test the statistical learning skills at birth, we recorded event-related brain responses of sleeping neonates while they were listening to a stream of syllables containing statistical cues to word boundaries.

**Results:**

We found evidence that sleeping neonates are able to automatically extract statistical properties of the speech input and thus detect the word boundaries in a continuous stream of syllables containing no morphological cues. Syllable-specific event-related brain responses found in two separate studies demonstrated that the neonatal brain treated the syllables differently according to their position within pseudowords.

**Conclusion:**

These results demonstrate that neonates can efficiently learn transitional probabilities or frequencies of co-occurrence between different syllables, enabling them to detect word boundaries and in this way isolate single words out of fluent natural speech. The ability to adopt statistical structures from speech may play a fundamental role as one of the earliest prerequisites of language acquisition.

## Background

Statistical learning can be described as the process of extracting the statistical properties of the data input. In speech, the statistical properties include the transitional probabilities between the different linguistic items, such as phonemes and syllables. Statistical learning of speech and sensitivity to social cues help infants in the rapid acquisition of their first language [1]. From 6 months of age, infants use computational strategies to learn the distributional patterns of sounds [2-4], the non-adjacent dependencies required to learn grammar [5,6], and the sequential probabilities [7,8], as well as the stress patterns [9], necessary to perform word segmentation. In the visual domain, infants as young as 2 months have been shown to learn statistical regularities in sequences of visual stimuli [10]. Although these skills are all based on computational mechanisms, they may, however, be based on very different underlying processes.

Recently, the mapping of different basic skills possibly used in language learning during the first year of life has undergone tremendous progress. For instance, it has been found that the skills necessary to discriminate different stress patterns in speech develop only after 4 months of age [11], even though newborns are able to process some rhythmic information in speech [12]. Still at the age of 7 months, infants use statistical rather than prosodic cues for recovering words from strings of syllables [13]. Furthermore, eight-month-old infants adopt these units as possible words in their native language [14]. Recent results also suggest that even newborn infants learn patterns of repetition in adjacent syllables located in tri-syllabic pseudowords [15]. However, the possibly very early capabilities of statistical learning that could exist as early as at birth have remained undiscovered. These skills could have an important contribution to the rapid language development that infants exhibit during the first months of life.

Kooijman, Hagoort, and Cutler [16], aiming at determining whether 10-month-old infants detect previously learned words from continuous speech, found that event-related potentials (ERPs) of 10-month-olds showed a greater negative deflection from 350 to 500 ms from the stimulus onset for familiar than unfamiliar words. The words in the experiment were learned in isolation, however. Furthermore, Sanders, Newport, and Neville [17], studying adult ERPs to three-syllable pseudowords hidden within a syllable stream, found an enhanced N400 amplitude and, for the high learners, an enhanced N100 amplitude for the pseudoword onsets after training. Even before training, the N100 amplitude was greater for the initial syllable of the pseudoword compared to that for the medial and final syllables, suggesting implicit learning of transitional probabilities in adult population. Additional evidence for the N100 and N400 ERP components as indices of adult statistical segmentation have recently been discovered [[18], Teinonen & Huotilainen: Implicit segmentation of continuous speech based on transitional probabilities: an MEG study, submitted].

In order to determine the availability of computational mechanisms for word segmentation at birth, we recorded ERPs in two successive experiments from 30 healthy sleeping neonates presented with a stream of syllables containing statistical cues to word boundaries. In the Experiment 1, we employed a paradigm previously used to determine statistical word segmentation in adults with magnetoencephalography [Teinonen & Huotilainen, submitted]. In Experiment 2, we replicated the results of the first experiment with minor modifications to the research design.

**Figure 1:**
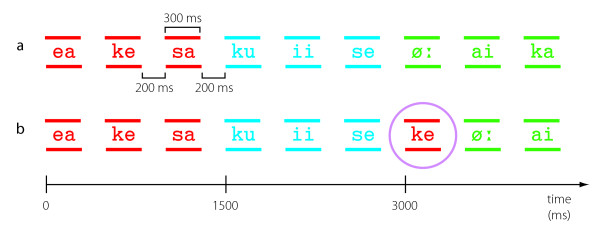
**Schematic of the experimental procedure**. (a) An excerpt of 9 syllables, i.e., 3 pseudowords from the speech stream within the first 15 minutes. (b) An excerpt of 9 syllables from the speech stream within the last 45 minutes; an unexpected syllable occurred every 2–4 words.

Ten different three-syllable pseudo-words were constructed so that each syllable was 300 ms in duration, with 200-ms breaks between the consecutive syllables and after the last syllable of a word. Each syllable belonged to one triplet only. The pseudo-words were played in a random order, but with equal transitional probabilities from a word to each of the other words (1/9). Thus, the sequence contained no morphological or prosodic cues to word boundaries (Fig. 1a). The resulting, seemingly random, stream of syllables was played for 15 minutes.

The sequences of syllables were acoustically controlled by placing syllables of a similar type, such as diphthongs, in all three positions of the words (see Methods). Thus, the acoustical properties of the syllables could not contribute to the learning of the word boundaries. Consequently, before learning the word boundaries, the responses to the initial syllable should not differ from those to the second and third syllables because of the similar acoustical properties of the syllables.

In order to assess the nature of the prospective processes responsible for detecting the word onsets, we also included unexpected syllables after 15 minutes of exposure. These unexpected syllables, occurring after every 2–4 words (Fig. 1b), consisted of the medial and final syllables of the previously-learned pseudowords and were presented alone between the intact pseudowords (i.e., as an "extra" syllable). One fourth of the deviant syllables were novel syllables not present earlier in the experiment.

In Experiment 2, the inventory of the syllables was same as in Experiment 1, but rearranged to create pseudowords with a different placement in the three syllable positions (i.e., the final syllables of the pseudowords from Experiment 1 were shifted to the initial position, see Tables 1 and 2). This was to ensure that no acoustic characteristics of the pseudowords could affect the effects measured. Additionally, the novel unexpected syllables were not used in Experiment 2, still keeping the frequency of unexpected syllables identical with Experiment 1 by increasing the number of unexpected medial and final syllables.

**Table 1:** Syllable stimuli in Experiment 1

		Pseudowords		Unexpected novel syllables
	**S1**	**S2**	**S3**	
1	/ø:	ai	ka/	/su/

2	/e:	ky	sæ/	/au/

3	/y:	sø	ki/	/ua/

4	/ea	ke	sa/	/ae/

5	/ui	si	o:/	/ei/

6	/ie	æ:	kæ/	/ue/

7	/sy	kø	eu/	

8	/so	ia	u:/	

9	/ku	i:	se/	

10	/ko	a:	iu/	

The syllables used in Experiment 1. Medial (S2) and final (S3) syllables were also used as unexpected syllables added between standard pseudowords.

**Table 2:** Syllable stimuli in Experiment 2

		Pseudowords	
	**S1**	**S2**	**S3**
1	/ka	ø:	ai/

2	/sæ	e:	ky/

3	/ki	y:	sø/

4	/sa	ea	ke/

5	/o:	ui	si/

6	/kæ	ie	æ:/

7	/eu	sy	kø/

8	/u:	so	ia/

9	/se	ku	i:/

10	/iu	ko	a:/

The syllables used in Experiment 2. The final syllables (S3) of Experiment 1 were shifted to become initial syllables (S1). Medial (S2) and final (S3) syllables were also used as unexpected syllables added between standard pseudowords. There were no novel unexpected syllables in Experiment 2.

## Results

### Experiment 1

**Figure 2:**
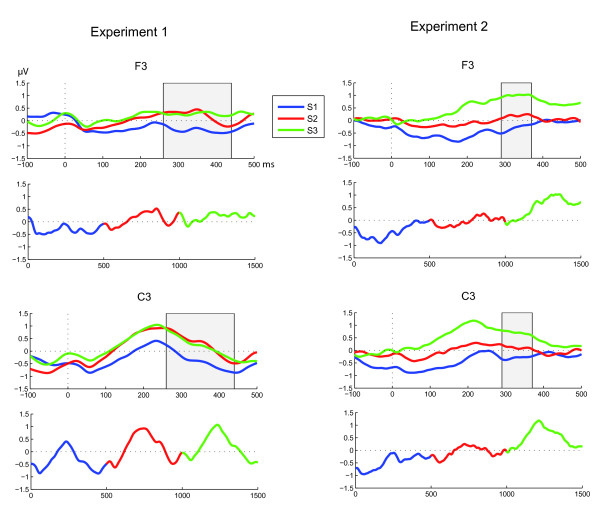
**Grand-averaged ERP amplitudes**. Grand-averaged event-related brain potentials (N = 15 in both experiments) to different syllables in the pseudowords and the whole pseudowords averaged over the whole experiments. The ERPs are time-locked to syllable onsets at time 0. The grey area indicates the time window of significant differences between the responses. In both experiments, there is a significant difference between the responses to S1 (blue) and S3 (green).

A three-way ANOVA [Syllable (S1, S2, S3) × Hemisphere (left, right) × Location (frontal, central, temporal, parietal)] was calculated on the average ERP amplitudes on consecutive 50 ms bins that shifted in steps of 10 ms (i.e., 0–50 ms, 10–60 ms, etc. until 450–500 ms). All the standard syllables (S1, S2, S3) except those in the triplets immediately following unexpected syllables were included in the averages. A significant main effect of Syllable on four consecutive bins was taken as evidence for differences between the responses for different syllables. This criterion was reached in the latency range of 260–440 ms relative to stimulus onset. The most significant bin, i.e., the one with a time range of 340–390 ms [*F*(2,24) = 5.563, *p *< .013], was selected as the latency of the effect. The response for S1 had a greater negative deflection than those for S2 and S3 (Figure 2). Post-hoc LSD tests confirmed that the response to S1 significantly differed from S2 (*p *< .006) and S3 (*p *< .034), but S2 did not differ from S3 (*p *> .975).

We found a significant main effect of Location [*F*(2,35) = 5.422, *p *< .006] caused by the absolute amplitude differences between the different scalp locations. However, the direction of the effect was similar in all four anterior positions (frontal, central, temporal, and parietal). Finally, we found a significant main effect of hemisphere [*F*(1,14) = 4.667, *p *< .049], with the amplitudes being overall more negative over the left hemisphere than over the right one. No significant interactions were found.

To further investigate the observed negativity at the word boundary, we included the initial syllables immediately following an unexpected syllable in the comparison, excluding the first 15 minutes of the data (i.e., when these syllables were not present). However, no significant main effects or interactions were found. This was likely due to the relatively low signal-to-noise ratio for the unexpected syllables due to a smaller number of repetitions compared to the within-word syllables, as well as the smaller number of repetitions for the within-word syllables, as the trials recorded during the first 15 minutes of the experiment were excluded. Consequently, the unexpected syllables were not analysed in Experiment 2.

### Experiment 2

A significant main effect of Syllable was reached in the latency range of 290–370 ms relative to stimulus onset. The most significant bin, i.e., 310–360 ms [*F*(2,28) = 3.454, *p *< .046], was selected as the latency of the effect. Similarly to Experiment 1, the negative deflection for S1 was larger in amplitude than those for S2 and S3. Post hoc LSD tests confirmed that the response to S1 significantly differed from that to S3 (*p *< .006) but not from that to S2 (*p *= .368), and the response to S2 did not significantly differ from that to S3 (*p *> .160).

We found a significant main effect of Location [*F*(2,34) = 4.575, *p *< .001] caused by the absolute amplitude the differences between different scalp locations. However, also in Experiment 2, the direction of the effect was similar in all four anterior positions (frontal, central, temporal, and parietal). We also found a significant interaction between Location and Hemisphere [*F*(2,29) = 5.461, *p *< .009], but no significant main effect of Hemisphere [*F*(1,14) = 3.814, *p *> .071].

Finally, we combined the data of the most significant bins in the two experiments and applied a four-way ANOVA [Experiment (1,2) × Syllable (S1, S2, S3) × Hemisphere (left, right) × Location (frontal, central, temporal, parietal)] to verify the replication of the results obtained in Experiment 1. We found no main effect of Experiment [*F*(1,14) = 1.450, *p *> .249] nor any interaction including Experiment (*p *> .186–.588). As expected, we found significant main effects of Syllable [*F*(2,28) = 6.578, *p *< .005] and Location [*F*(2,33) = 11.579, *p *< .00007]. There was also a significant main effect of Hemisphere [*F*(1,14) = 8.663, *p *< .011] and an interaction of Hemiphere and Location [*F*(2,25) = 4.142, *p *< .032], with the responses over the left hemisphere being larger especially at the central electrode site than those over the right hemisphere. No other main effects or interactions were found. LSD tests of the combined data confirmed that the response for S1 was larger in amplitude than those to S2 (*p *< .045) and S3 (*p *< .002), but the response to S2 did not differ from that to S3 (*p *> .210).

## Discussion

We recorded ERPs of 15 sleeping newborn infants, presented with repeating pseudowords containing occasional unexpected syllables. We also recorded a further set of data of 15 additional infants, because using a novel research design, it was important to replicate the findings. Furthermore, one could argue that the novel unexpected syllables used in Experiment 1 could provide additional information with respect to the location of the word boundaries. Thus, the unexpected novel syllables were not used in Experiment 2. As the unexpected syllables may considerably contribute to the perceptive processes of the infants, however, it was important to preserve the unexpected medial and final syllables in Experiment 2 to enable the direct comparison between the results of these two experiments.

In Experiment 1, we found that the neonate brain began to respond specifically to the pseudowords embedded in a seemingly random stream of syllables. A larger negative deflection was detected in the response to the initial syllable S1 compared with those to the other two syllables, S2 and S3. In Experiment 2, we found differences at a similar latency between the responses to the different syllables. A larger negative deflection was detected in the response to S1 compared to that in the response to S3.

Because the corpus used in the experiment consisted of 10 different words, it was highly unlikely to remain in auditory sensory memory during the experiment due to its limited capacity and interference caused by continuous stimulation [19]. Furthermore, due to the large number of different syllables and the acoustically balanced positioning of the syllables, learning could not be specific to any single items. Therefore, the results demonstrate efficient segmentation of continuous speech into distinct units solely on the basis of the statistical properties. The properties used could either be the transitional probabilities between consecutive syllables, a strategy that 8-month-old are able to use [8], or they may be based on learning the frequencies of co-occurrence of consecutive syllables, higher within words than between them.

One could also argue that the difference between the frequencies of individual S1 syllables and S2 or S3 syllables (Table 3) could have provided an additional cue for distinguishing these syllables from each other. However, as even the largest differences in frequency (occurring within the last 45 minutes of Experiment 2 between the 10 S1 syllables, the frequency of each being 3.000%, and the 20 S2/S3 syllables, the frequency of each being 3.500%) were very small, it is unlikely that this subtle variation could have been detected or in any way utilised by the infants within the limited time of the experiment.

**Table 3:** Syllable stimulus frequencies

	***Experiment 1***		***Experiment 2***	
	
	Last 45 minutes only	Entire experiment	Last 45 minutes only	Entire experiment
	
S1 (10 different)	3.000%	3.083%	3.000%	3.083%
	
S2 (10 different)	3.375%	3.365%	3.500%	3.458%
	
S3 (10 different)	3.375%	3.365%	3.500%	3.458%
	
Novel syllables (6 different)	0.417%	0.313%	0%	0%

The frequencies of different acoustic syllables (36 different in Experiment 1, 30 different in Experiment 2; see Tables 1 and 2) shown in percentages for the last 45 minutes (the part that included unexpected syllables) and the entire experiments. During the first 15 minutes (the part without unexpected syllables), the probabilities of S1, S2, and S3 syllables were equal (i.e., 3.333%).

The results obtained in Experiment 2 did not replicate the significant difference between S1 and S2, found in Experiment 1. It is reasonable, however, to assume that the difference between S1 and S3 is more prominent than that between S1 and S2, as the grand-averaged responses to the whole pseudowords show an overall positive trend (see Fig. 2). As the neonate ERPs typically have a very large inter-individual variation, it could result in the less prominent difference between S1 and S2 being evident in the ERPs of some individuals only. Additionally, the syllable stimuli used for S1 and S3 in Experiment 1 were used for S2 and S1, respectively, in Experiment 2. Thus, the difference between the responses to S1 and S3 in Experiment 1 and the lack of a difference between the responses to S2 and S1 in Experiment 2 indicate that the observed effects were not caused by any acoustical characteristics of the syllables. It should also be noted that when the data from Experiments 1 and 2 were combined, the response to S1 showed a larger negative deflection than those to both S2 and S3.

The observed differences between the responses to S1 and S3 may be due to various reasons. Firstly, the lack of predictability caused by the smaller transitional probability at the word boundary could be processed differently from the way of how completely predictable within-word transitions were processed. This could be seen as anticipatory processes emerging during the processing of the final syllable or even before it. On the other hand, the onset response could be interpreted as a specific reaction to the beginning of a new word. Such a word onset response may be explained by pseudowords being distinguished and memorised in the brain or by cerebral memory traces of the relationships between syllable pairs within the words.

The underlying processes need to be studied further before any final conclusions on the nature of the learning mechanisms can be made. The differences seen between the responses to the initial syllables and those to the final syllables of the words suggest that these syllables may be the key elements of detecting the word boundaries. It is also interesting to note that in a similar task, an enhanced negativity for the onset syllable is also observed in the adult N100 and N400 responses [[17,18], Teinonen & Huotilainen, submitted].

## Conclusion

We found that newborn infants are sensitive to the statistical properties of a stream of speech. More specifically, our results show that even the neonatal brain effectively segments word-like units from a stream of syllables using only the transitional probabilities or frequencies of co-occurrence between the syllables. These results hence suggest a role for statistical learning in the early acquisition of words.

## Methods

### Participants

Fifteen healthy full-term neonates (8 boys) with a mean gestational age of 40 weeks and 1 day (range 38 weeks 1 day – 42 weeks 2 days) and a mean birth weight of 3.713 kg (range 2.610–4.588 kg) participated in Experiment 1. The Apgar score range was 8–10. The neonates were tested between 0.5 and 2 days after birth. Five out of 15 infants were born by Cesarean section. Additional fifteen healthy full-term neonates (7 boys) with a mean gestational age of 40 weeks and 1 day (range 37 weeks 5 days – 41 weeks 4 days) and mean birth weight of 3.574 kg (range 2.900 – 4.330 kg) participated in Experiment 2. The Apgar score range was 9–10. The neonates were tested between 0.5 and 2 days after birth, except for one who was tested 6 days after birth. The Ethics Committee for Paediatrics, Adolescent Medicine, and Psychiatry, Hospital District of Helsinki and Uusimaa approved the study protocol, and a written informed consent was obtained from one or both parents of the neonates.

Only the brain activity recorded while infants were in active sleep was used in the analysis in order to reduce the variation caused by the altering arousal states. Active sleep was defined as a behavioural state in which the infant's eyes were closed or nearly closed, eye movements were apparent, breathing was irregular and minor muscle movements, small movements of extremities and even large generalized movements occurred intermittently [20]. The infants were in active sleep 40–80% of the time in Experiment 1 and 50–90% of the time in Experiment 2.

### Stimuli

Ten different three-syllable pseudo-words were constructed so that each syllable was 300 ms in duration, separated by 200 ms of silence throughout the entire stream. Each syllable belonged to one triplet only. The pseudo-words were played in a random order, but with equal transitional probabilities from a word to each of the other words (1/9). Thus, the sequence contained no morphological or prosodic cues to word boundaries (Fig. 1a). The resulting, seemingly random, stream of syllables was played for 15 minutes.

Speech stimuli were cut from natural utterances of a female speaker recorded in an anechoic chamber. At least 7 repetitions for each of the 36 syllables used in the experiment were recorded. Clear samples with an approximately equal pitch were selected for the experiment. The syllables were cut to 300 ms in duration. Four different types of syllables were used: /k/ + vowel, /s/ + vowel, long vowel, and diphthong. The syllables were chosen so that the fundamental frequency of the voice remained relatively stabile throughout the syllables.

Ten pseudo-words (Table 1) were constructed out of 30 of the syllables so that each word consisted of 3 syllables of different types. All types of syllables were present in every position of the words equally many (2–3) times and every type of syllable followed every other type of syllable equally many (1–2) times in order to avoid any regularities in the syllable sequence besides the transitional probabilities between the syllables. This ensured that the differences in the brain responses to the different syllable types caused by the differences in the acoustic properties of the four syllable types did not affect the averaged responses. Additionally, the words used in Experiment 2 were modified so that the final syllables of the words used in Experiment 1 were shifted to the initial position (see Tables 1 and 2) to eliminate the possibility of stimulus-specific effects. The syllable stream was created so that every word followed every other word equally frequently, keeping the word order otherwise random. Thus, the pseudowords could not be separated from the syllable stream without learning the transitional probabilities between the consecutive syllables.

After the first 15 minutes of the experiment, one of the medial or final syllables, or in Experiment 1, in one fourth of the cases, one of the 6 syllables not present earlier in the experiment (Table 1) was added after every 2–4 words. The entire experiment lasted 60 minutes. All medial and final syllables were used as unexpected syllables, and the added medial and final syllables were equiprobable (see Table 3 for overall frequencies of the different syllables). In Experiment 1, in about 1/10 of the unexpected syllables, the unexpected syllable was present in the just preceding word. These cases were excluded from the averages. In Experiment 2, such cases did not exist.

### EEG recording and data analysis

The EEG was recorded in a quiet room from 8 standard electrode sites spanning the scalp. Single-use electrodes were used for recording the EEG from the scalp (electrodes F3, F4, C3, C4, T3, T4, P3, and P4 according to the 10–20 system), mastoids, and EOG from the canthus and below the eye. Linked mastoids were used as a reference. Sounds were presented through two loudspeakers placed 20 cm from both sides of the infant's head. The EEG had a sampling rate of 250 Hz in Experiment 1 and 500 Hz in Experiment 2, and was digitally filtered (passband 0.2–30 Hz).

The measurement was divided into blocks of 5 minutes according to the sleep stages of the infants. The active sleep blocks were further divided into epochs of 500 ms, i.e., the duration of one syllable including the silent intervals after the syllables. Epochs with artifacts exceeding ± 150 μV were discarded. The average number of the remaining trials per subject was 1036 for S1, S2, and S3 in Experiment 1 and 1141 in Experiment 2. No baseline correction was used, as the stimulus rate was rapid relative to the neonatal brain responses, and thus the responses for successive stimuli were likely to partially overlap or occur just before the next stimulus. Consequently, there was no natural baseline period that would not contain any stimulus-related activity. Huynh-Feldt correction was used in all the ANOVAs to assess significance.

## Authors' contributions

MH and TT conceived of and designed the study. TT, PA, and MH prepared the stimuli. MH, TT, and VF coordinated the EEG recordings. TT performed the data analyses. TT, MH, and RN interpreted the results. All authors participated in the writing process and read and accepted the final version of the manuscript.
